# The Role of the Physician-Scientist in Our Evolving Society

**DOI:** 10.5041/RMMJ.10063

**Published:** 2011-10-31

**Authors:** Michael R. Rosen

**Affiliations:** Gustavus A. Pfeiffer Professor of Pharmacology and Professor of Pediatrics; Director, Center for Molecular Therapeutics, Columbia University Medical Center, New York, NY, USA

**Keywords:** Academic medicine, developing new faculty, review and funding of research

## Abstract

The physician-scientist represents the medical-scientific version of the “triple threat” athlete. Yet, in medicine as in sports, specialization and business are ever more in the forefront. As the field of medicine evolves, it is likely that the role of the physician, the scientist, and the physician-scientist will continue to change. Whether this is for the good or bad will only be known in hindsight.

## INTRODUCTION

As a physician-scientist who has been “in the field” for nearly 50 years, I have found myself reflecting on the landscape that greeted me in the 1960s and the one I see in the second decade of a new millennium. To say I am perplexed and worried would be to understate. To explore the root of this malaise, I shall review who and what the physician-scientist was and is, and what his/her prospects appear to be. My intent is not to do this in a “personal” way. Rather, I attempt to achieve some objectivity by presenting and commenting on the words and the thoughts of others.

## WHAT IS A PHYSICIAN-SCIENTIST?

There are varied definitions of “physician-scientist”, but the two I prefer are as follows:

“… individuals holding an MD or MD/PhD degree who perform biomedical research of any type as their primary professional activity …”^1^ and, alternatively, “… those considered to be clinicians by physiologists, biochemists, and immunologists; and considered to be physiologists, biochemists, or immunologists by most clinicians …”^2^ Within those general frameworks, as discussed by Zemlo et al.,^2^ the physician-scientist is one who …

“1) is trained to ask clinically relevant questions in a health research environment that lead to development of research linking basic and clinical sciences; 2) transforms clinical observations into testable research hypotheses and translates research findings into medical advances; 3) assures excellence in medical education … teaching students that the basis of medicine is science and that scientific rigor should apply to patient care as well as research; and 4) has specialized perspectives required to lead evolving fields such as genetic medicine, pharmacogenetics, and bioinformatics.”

**Figure 1:**
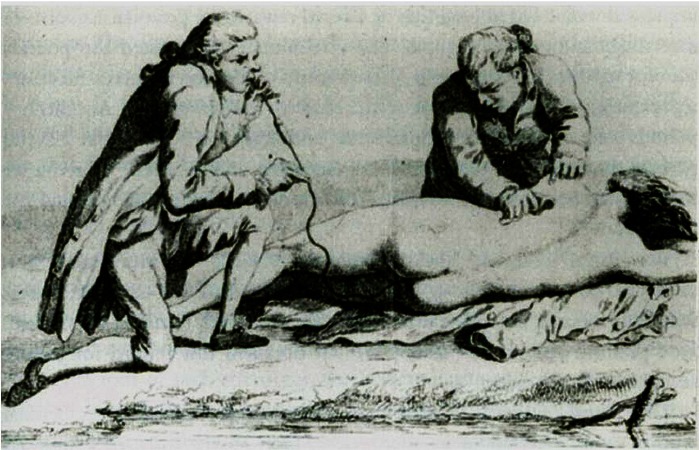
**A 1700s approach to resuscitation.** Electrodes are applied to the chest, while smoke is blown up the anus. From Akselrod et al., in: Efimov IR et al., eds. Cardiac Bioelectric Therapy: Mechanisms and Practical Implications. Springer; 2009,^4^ by permission.

Who and what the physician-scientist should be has occupied the attention of thought-leaders for years: as reviewed by Ginsburg,^3^ from 1939 to 2001 the subject most frequently discussed in addresses by all heads of the American Society for Clinical Investigation has been the future of the physician-scientist. Of course in defining the physician-scientist we are confronted by an ever-changing image. For example, the physician-scientist in the 1700s was a bewigged, knickerbocker-clad individual who might administer to a syncopal patient by applying electrodes to the chest (still done in modern times with better equipment) while blowing smoke up the anus (Figure 1).^4^ The latter approach, while pharmacologically sound (administering a stimulant via an absorptive mucosa), appears not to be generally used today, although those faced with administrative realities of modern institutions might argue otherwise.

Finally, there is the argument of whether a physician-scientist is an MD or an MD-PhD who does research, whether that research can be basic or “translational”, and/or whether it should be clinical or at least clinically impacting. With regard to the latter it has been asked whether clinically associated research gives us the biggest return with regard to improving health care. My favorite response to this question was provided years ago by Comroe and Dripps:^5^ they and a team of consultants evaluated the contributions to research leading to what were judged the 10 top advances in cardiopulmonary medicine over a roughly 30-year period ending in the 1970s. They concluded that 41% of all work that was essential for later clinical advance was not clinically oriented at the time it was done, that the scientists responsible for these key articles sought knowledge for its own sake, and that clinical advance requires different types of research and development and not one to the exclusion of the other. In a later publication^6^ Comroe stated:

“Our data compel us to conclude that a generous portion of the nation’s biomedical research dollar should be used to identify and then provide long term support for creative scientists whose main goal is to learn how living organisms function, without regard to the immediate relation of their research to specific human diseases; and that basic research pays off in terms of key discoveries almost twice as handsomely as other types of research and development combined.”

Comroe and Dripps^5^ also recommended means for tracking the effectiveness of research, stating that …

“independent, highly competent groups be established with ample, long term support to conduct and support retrospective and prospective research on the nature of scientific discovery, to analyse the causes of long and short lags between discovery and clinical application and to suggest and test means of decreasing long lags, and to evaluate present and proposed mechanisms for the support of biomedical research and development.”

As evaluated later by Smith:^7^

“The real lesson from Comroe and Dripps … is that we need to research research so that we can allot funds in a more intelligent and less empirical and anecdotal way … The lessons from Comroe and Dripps have not been learnt to any great extent by those funding medical research.”

## WHAT IS THE UNIVERSE THE PHYSICIAN-SCIENTIST INHABITS?

The limits of the universe inhabited by the physician-scientist are defined largely by time and money. In a *Journal of Clinical Investigation* editorial, Andrew Marks stated:^8^

“Historically, physician-scientists have had dual roles in caring for patients and in performing investigative research that could potentially lead to new diagnostics and therapeutics. Physician-scientists conducted teaching rounds in the hospital … and were often avidly pursued as the most important sources of new knowledge for trainees.

“… Now physician-scientists are rarely seen in the hospital; they are most often spotted at their desks tapping out yet another grant application. Most struggle to find the time to mentor students and clinical trainees, let alone to care for patients, even though these interactions are often the motivating forces for scientific creativity.”

These statements accompany a number of realities, as follows: Data collected by the American Medical Association from 1960 through 2005 show a major rise in the number of US physicians engaged in patient care while those involved in research and in teaching have been flat. Considering number of faculty in medical school departments and National Institutes of Health (NIH) grants funded over roughly the same period, PhDs are increasing in number, MDs are decreasing, and the number of MD-PhDs is flat. Of interest as well is that despite the efforts of NIH to fund young investigators, the trend over a 40-year period has been for the average age of research grant RO-1 recipients to increase, such that in 2005 approximately 35% of RO-1 principal investigators were over 50 years of age (as compared to about 22% 20 years earlier).

Barbara Weber has summarized the time and money issues confounding the physician-scientist as follows:^9^

“Three [critical] issues in academic medicine [are] having a serious negative effect on the pace and quality of academic … investigation. 1) The cost to creative time of ‘feeding the beast’ of the academic bureaucracy; 2) the innovation-squelching nature of the current peer review system; 3) the loss of physicians with a passion for clinical investigation as the leaders of academic medical centers.”

In this piece, she notes that a shift away from academic leaders being the leaders of academic medical centers has occurred because of the time drain of “feeding the beast” and that a premium is put on the financial bottom-line to the point that clinical investigation is no longer central to the mission statements of academic medical centers. She also notes that leadership is ever more in the hands of individuals who are not so much scientific leaders and role models as they are “businessmen”, who wind up being “adversaries to many faculty because of the business models under which they … operate”.^9^ this has been stated differently by Gary Koretzky: “It now seems that MD/MBAs may be more valued than MD/PhDs”.^10^

With regard to the judgment of research in an atmosphere in which funds are ever-more constrained, Weber offers advice that is sound, albeit rarely listened to these days.^9^ She states that reviewers should: “1) Focus on the big picture, and not … worry too much about details. 2) Ask, ‘Is this an important question, with plausible hypotheses?’ 3) Never say things like ‘overly ambitious’ or ‘it may not work’.” A neat coda is offered to this statement by Terry Strom who notes: “If we knew it would work it wouldn’t be research.”

With regard to the impact of NIH funding on the physician-scientist’s universe, it is not as if there isn’t a good deal of money in NIH. Rather, especially after the doubling of the NIH budget, the issue at present is in large part allocation of funds. A good illustration of how not to invest research funds – whether to physician-scientists or to scientists in general – is provided by the 2009 American Recovery and Reinvestment Act (ARRA). As part of an economic stimulus package this provided $10.4 billion to NIH. The Institute’s web site highlighted the ARRA funds as follows: “NIH’s two-year infusion of ARRA funds will empower the nation’s best scientists to discover new cures, advance technology, and solve some of our greatest health challenges.”^11^ The statement is an unfortunate, hard-sell advertisement of the potential benefits of the funding, rather than reflecting any sober assessment of what is really required to make scientific progress aimed at bettering health care. Consider that the review process for ARRA grants was tailored as little more than a questionnaire for reviewers to fill out and that at least one grant was funded in every US State plus Guam and US Virgin Islands. This was not the funding of science for the sake of supporting the best and the brightest: that likely could have been done better by simply giving the monies to NIH to support the best research being submitted and reviewed by traditional means. Indeed the only conclusion that one can reach *re* the ARRA grants process is that politics trumps science. This should not be taken to mean that some outstanding research was not funded by the ARRA process; it does mean that the entire process for evaluating research was side-tracked for political imagery and for short-term economic advantage.

## HOW TO ADVANCE THE FIELD

This issue has been addressed by a number of individuals, and both Zemlo^1^ and Marks^8^ have come up with ideas that are summarized here.

1. School curriculum emphasis of the importance of biomedical research as a foundation for the scientific principles that govern the practice of medicine.
2. A national program for medical school debt forgiveness for physicians who receive rigorous research training and pursue research careers.
3. Substantial expansion of support for the training and mentoring of physician-scientists by NIH and other appropriate foundations.
4. Development in Academia of favorable institutional cultures to support physician-scientists throughout their careers.
5. Collection of additional information to define the problem further and to monitor the outcomes of corrective efforts.
6. Redefinition of the roles of clinicians and clinician-scientists within the medical centers.

In attempting to accomplish the above, the following statement is worth remembering: “Assistant professors are hired based on their scientific research accomplishments but their success as faculty members is very much related to their ability to manage a small business.”^12^ Another view of the current situation states:

“We in universities and laboratories frequently are exhorted to run our institutions more like businesses. It is fair to note that not every business is brilliantly run, but that is not the essence of why such advice is misguided. A business makes products, sells services, strives for profit. A university or laboratory exists to seek truths, test ideas, transmit knowledge and the habits of free inquiry. Both sets of goals may be noble. They are different!”^13^

Putting all this together, I fear that unless and until we permit our physician-scientists to get back to the mainstream of their profession, we will continue to have a system that operates in the interface of business and science and sacrifices both stability and potential greatness in the process. Despite this fear, will a hybrid approach that marries business and science advance our field? No doubt it will. Will the rate of advancement be as rapid as that which has occurred using earlier models? We’ll likely never get to test this accurately. What I mourn as I see the union of business and science advance is the loss of the lone investigator, one with a small lab and big ideas who is enabled to explore the limits of his/her intellect in an environment that appreciates, encourages, and supports his/her approach. Putting it another way: intellect for its own sake has its place; business has its place – and while interfacing them is a reasonable goal, I’d rather see them divorced than married.

## References

[1] Zemlo TR, Garrison HH, Partridge NC, Ley TJ (2000). The physician-scientist: career issues and challenges at the year 2000. FASEB J.

[2] Starr I (1940). Functions and dysfunctions of learned societies: proceedings of the thirty-second annual meeting of The American Society for Clinical Investigation. J Clin Invest.

[3] Ginsburg D (2002). The history and evolution of the ASCI: déjà vu all over again. American Society for Clinical Investigation. J Clin Invest.

[4] Akselrod H, Kroll MW, Orlov MV, Efimov IR, Kroll MW, Tchou PJ (2009). History of defibrillation. Cardiac Bioelectric Therapy: Mechanisms and Practical Implications.

[5] Comroe JH, Dripps RD (1976). Scientific basis for the support of biomedical science. Science.

[6] Comroe JH, Dripps RD (1977). Biomedical research pays off. Pediatrics.

[7] Smith R (1987). Comroe and Dripps revisited. Br Med J (Clin Res Ed).

[8] Marks AR (2007). Physician-scientist, heal thyself. J Clin Invest.

[9] Weber BL (2007). In the palace of the sultan: 2007 American Society for Clinical Investigation Presidential Address. J Clin Invest.

[10] Koretzky GA (2001). The future of the ASCI (American Society for Clinical Investigation): a lesson from 2000 presidential election. J Clin Invest.

[11] Office of Science Policy Analysis, NIH. Available at: ospa.od.nih.gov/advances (accessed April 2011)

[12] Aschwanden C (2008). Managing to excel at science. Cell.

[13] Quigg C (2003). Closing remarks at the Illinois Mathematics and Science Academy Dialogue: Ethical awareness for tomorrow’s leaders.

